# Quantifying the Reconfiguration of Intrinsic Networks during Working Memory

**DOI:** 10.1371/journal.pone.0106636

**Published:** 2014-09-05

**Authors:** Jessica R. Cohen, Courtney L. Gallen, Emily G. Jacobs, Taraz G. Lee, Mark D'Esposito

**Affiliations:** 1 Kennedy Krieger Institute, Baltimore, MD, United States of America; 2 Department of Neurology, Johns Hopkins University School of Medicine, Baltimore, MD, United States of America; 3 Helen Wills Neuroscience Institute, University of California, Berkeley, CA, United States of America; 4 Department of Psychiatry, Harvard Medical School, Boston, MA, United States of America; 5 Department of Psychiatry & Medicine, Division of Women's Health, Brigham and Women's Hospital, Boston, MA, United States of America; 6 Department of Psychological and Brain Sciences, University of California Santa Barbara, Santa Barbara, CA, United States of America; 7 Department of Psychology, University of California, Berkeley, CA, United States of America; University Of Cambridge, United Kingdom

## Abstract

Rapid, flexible reconfiguration of connections across brain regions is thought to underlie successful cognitive control. Two intrinsic networks in particular, the cingulo-opercular (CO) and fronto-parietal (FP), are thought to underlie two operations critical for cognitive control: task-set maintenance/tonic alertness and adaptive, trial-by-trial updating. Using functional magnetic resonance imaging, we directly tested whether the functional connectivity of the CO and FP networks was related to cognitive demands and behavior. We focused on working memory because of evidence that during working memory tasks the entire brain becomes more integrated. When specifically probing the CO and FP cognitive control networks, we found that individual regions of both intrinsic networks were active during working memory and, as expected, integration across the two networks increased during task blocks that required cognitive control. Crucially, increased integration between each of the cognitive control networks and a task-related, non-cognitive control network (the hand somatosensory-motor network; SM) was related to increased accuracy. This implies that dynamic reconfiguration of the CO and FP networks so as to increase their inter-network communication underlies successful working memory.

## Introduction

Humans are remarkably adaptable due, in part, to the flexibility with which different brain regions and functional networks are engaged when confronted with a constantly changing environment. It has been hypothesized that the pattern of interactions across neural regions is critical for cognition [1], [2]. These theories emphasize the existence of rapid and transient changes in connections across neurons due to changes in one's current environment. It has been demonstrated that such rapid changes in neural communication are detectable using functional MRI (fMRI), as measured both when participants are at rest [3], [4] and when they are engaged in a specific task [5], [6].

In the current study, we sought to elucidate how brain networks adaptively change in a rapid, transient manner in response to changing cognitive demands. Network organization was quantified using functional connectivity analyses with fMRI data. There is currently a large focus on studying low-frequency (<.1 Hz) blood-oxygen-level-dependent (BOLD) fMRI fluctuations that are observed while an individual is at rest. Brain regions that are functionally connected at rest are thought to reflect an intrinsic state, with the implication that intrinsic connectivity may underlie or predict numerous qualities of an individual, from cognitive ability [7]–[9] to disease state [10] to age [11]. Such intrinsic networks are reliable both within individuals [12] and across populations [13].

Several intrinsic networks that have been reliably detected are thought to be related to cognitive control, or the ability to flexibly adapt thoughts and behavior in a goal-directed manner through processes such as selective attention, maintenance, updating, and the inhibition of irrelevant information. Many intrinsic cognitive control networks have been identified; in the current study we focused on two such networks: the cingulo-opercular (CO; thought to underlie task-set maintenance and tonic alertness) and the fronto-parietal (FP; thought to underlie adaptive, trial-by-trial updating) networks [7], [14]. The purported roles of these networks in cognitive control have been inferred mainly from previous studies examining the functions of individual regions within each network, as opposed to examining network interactions (for a rare exception, see: [15]).

While most research probing intrinsic networks has focused on the resting state, some studies have investigated intrinsic network organization during task performance. Overall, whole-brain intrinsic network organization as measured during rest seems to remain fairly similar when participants are engaged in cognitive tasks [16], although the degree of similarity is different for different areas of the brain (i.e., multimodal association areas have been found to display more similar functional connectivity with the rest of the brain during task as compared to rest than subcortical and primary motor areas [17]). When honing in on task-relevant intrinsic networks, there is some evidence that they encompass more regions during task as compared to rest [18].

No existing study examining intrinsic networks during a task has focused on specific cognitive control networks, or probed how intrinsic network reconfiguration during tasks contributes to performance. The goal of the current study, therefore, was three-fold: 1) to investigate functional connectivity of the intrinsic CO and FP networks during cognitive control; 2) to determine how these groups of regions, acting as networks, reconfigure from intrinsic connectivity patterns when confronted with a specific cognitive environment; and 3) how that reconfiguration relates to cognitive control ability. Specifically, we quantified how intrinsic network connectivity changed during a task that tapped working memory (WM) function. Based on workspace theories of cognition [19], we theorized that these two intrinsic networks, which are distinct at rest [7], would become more integrated under conditions requiring increased cognitive control. We chose to probe this pattern of reconfiguration during WM because of previous findings using magnetoencephalography that the entire brain becomes more globally efficient, and therefore more integrated, with increased WM demands [20].

## Materials and Methods

### Ethics Statement

All procedures were approved by the University of California, Berkeley Committee for the Protection of Human Subjects. All participants provided written informed consent.

### Participants

39 healthy young adults (age range 18–29, 30 female) recruited for three separate studies (two of which have been published: [21], [22]) were included in this analysis. Participants were excluded from the original studies for any history of neurological or psychiatric disorders, an episode of loss of consciousness, use of psychotropic drugs, a history of substance abuse, MRI contraindications, or, for Study 1, abnormal or infrequent menstrual cycles or use of a hormonal birth control. All participants completed more than one session; only data from their first session were included here.

The procedure of the first session differed for each of the three studies. Participants of Study 1 completed two cognitive control tasks, always in the same order: first an N-back WM task with 0-, 2-, and 3-back blocks, then the Selective WM task analyzed here. It is important to note that data from the N-back task were analyzed using the same methods as detailed below, with equivalent results. They are not discussed further because of the small number of participants with complete N-back data (n = 15) and therefore limited power to detect significant results. After the cognitive control tasks, participants completed functional localizers and a resting state scan. Participants of Study 2 completed a resting state scan, followed by the Selective WM task analyzed here, and concluded the session with a second resting state scan and a functional localizer. Participants of Study 3 completed only the Selective WM task. While the research questions were different for each of the three studies, it is not expected that the specific procedures influenced functional connectivity during or performance on the Selective WM task.

For the current analyses, participants were included only if they met the following criteria: complete datasets (i.e., no incomplete scans or missing behavior), minimal motion during fMRI scans, acceptable task performance, and functional connectivity values that were not outliers. Data was considered complete only if both behavioral and neuroimaging data existed for at least 3 complete runs of each task condition (see below task description for details). Minimal motion was defined as no spikes greater than 2 mm. Acceptable task performance was defined a priori as: 1) response rate of at least 85%; and 2) accuracy or median response time (RT) within two standard deviations of the group mean in any condition. Outliers for functional connectivity values were defined a priori as average connectivity greater than two standard deviations from the group mean.

These inclusion criteria resulted in 15 participants from Study 1, 16 participants from Study 2, and 8 participants from Study 3, for a total of 39 participants.

### Experimental Task

**Figure 1:**
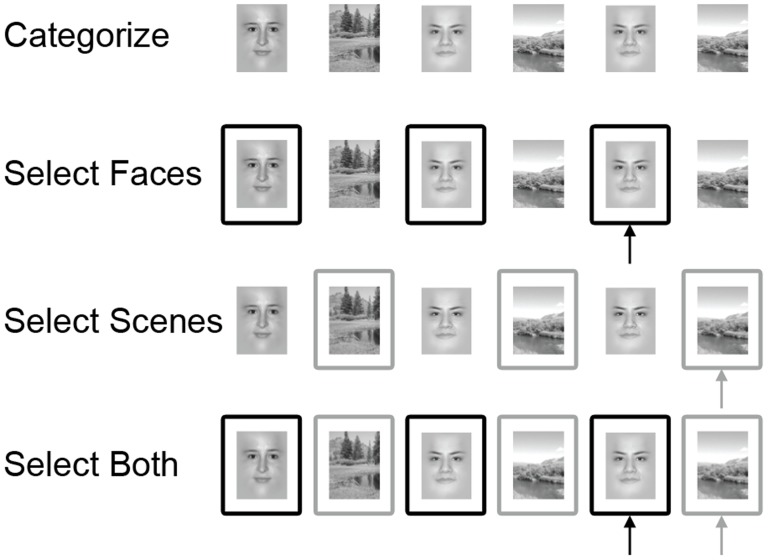
Experimental design of the Selective WM task. All blocks consisted of sequences of 20 stimuli (10 faces and 10 scenes in pseudo-random order), presented one at a time. Black and gray boxes around the stimuli are for illustrative purposes and highlight which trials were relevant for each block. Arrows point to match trials.

Data from a Selective WM task were analyzed here. The task consisted of 16 (Study 1) or 20 (Studies 2 and 3) runs of approximately two minutes each (Figure 1). It was a modified 1-back task with high demands on selective attention. Participants were presented with a series of face or scene images that appeared sequentially. Each image was on the computer monitor for 600 ms. There was a jittered delay between consecutive images (randomly ordered: 2400, 4400, or 6400 ms) to allow for event-related analyses. Each run contained 20 trials (10 faces and 10 scenes in pseudo-random order). There were four task conditions that differed in WM load: ‘Categorize’, ‘Select Faces’, ‘Select Scenes’, and ‘Select Both’. Participants responded to each stimulus with one of two buttons using the index and middle fingers of their right hand. During Categorize runs, participants indicated whether the stimulus was a face (left button press) or a scene (right button press). During Select Faces runs, participants were instructed to attend only to face stimuli and indicated whether each face matched the previous face (right button press). Non-match face stimuli and unattended trials (all scenes) were responded to with a left button press. During Select Scenes runs, participants were instructed to attend only to scene stimuli and indicated whether each scene matched the previous scene (right button press). Non-match scene stimuli and unattended trials (all faces) were responded to with a left button press. Because WM load was comparable for Select Faces and Select Scenes runs, these were combined and referred to as ‘Select Relevant’ runs. During Select Both runs, participants were instructed to attend to both the face and scene stimuli and indicated whether the current stimulus matched the previous stimulus of the same type (i.e., whether the current face matched the previous face and whether the current scene matched the previous scene; right button press). All non-matches were responded to with a left button press.

### fMRI Data Acquisition and Processing

Imaging data were collected on two identical 3-Tesla Siemens MAGNETOM Trio whole-body MR scanners (data for Study 1 were collected at the University of California, San Francisco Neuroscience Brain Imaging Center; data for Studies 2 and 3 were collected at the University of California, Berkeley Brain Imaging Center). A 12-channel head coil was used for all studies. Whole-brain functional data (1824 volumes for Study 1; 2280 volumes for Studies 2 and 3) were acquired using a T2*-weighted echo-planar imaging (EPI) pulse sequence using GRAPPA with acceleration factor 2 for Study 1 (TE = 27 ms), and no parallel imaging for Studies 2 (TE = 24 ms) and 3 (TE = 32 ms). All studies collected 18 axial slices parallel to the AC-PC line (interleaved for Studies 1 and 3; descending for Study 2). Slices were 5 mm thick for Studies 1 and 2, and 3 mm thick for Study 3 (interslice distance = 0.50 mm, TR = 1000 ms, FA = 62°, matrix 64 × 64 for all studies). The field of view was 225 mm for Studies 1 and 2 and 230 mm for Study 3. A high-resolution T1-weighed structural 3D MP-RAGE was also acquired for all studies (160 slices, slice thickness 1 mm, TR = 2300 ms, TE = 2.98 ms, FA = 9°, matrix 256 × 256, field of view 256 mm). An LCD projector back-projected visual stimuli onto a screen mounted to the RF coil. E-Prime software (Psychology Software Tools, Pittsburgh, PA) was used to present stimuli and record responses and latencies via a fiber-optic motor response recording device.

Processing was carried out using FSL 4.1 (FMRIB's Software Library: www.fmrib.ox.ac.uk/fsl). Images were corrected for motion using MCFLIRT and the brain was extracted from the skull using BET.

### Univariate fMRI Data Analysis

The univariate analyses were conducted under the assumptions of the general linear model (GLM) in FSL 4.1 using FEAT (version 5.98). Images were spatially smoothed with a 5 mm FWHM isotropic Gaussian kernel and temporally filtered with a high-pass filter (100 sec cutoff). Time-series statistical analyses were carried out using FILM with local autocorrelation correction (Gaussian-weighted least squares straight line fitting, with sigma = 33.0 s). Individual events were modeled for correct face and scene stimuli separately for each block, using two regressors: one of constant duration (the duration was defined as the average correct RT for that event-type) and one that was RT-modulated (the duration of each event was the RT for that particular trial). In this manner, all effects that were due purely to differences in RT could be controlled for. Each event was defined as a delta function. All regressors of interest were created by convolving each event of interest with a canonical (double-gamma) hemodynamic response function [23]. In addition to regressors of interest, incorrect and missed trials, estimated motion parameters, and temporal derivatives for each regressor were included as nuisance regressors. Linear contrasts were performed for the comparison of interest. To determine whether regions involved in each contrast were specific to the conditions examined or general to cognitive control, we conducted two cognitive control-related contrasts. First, we examined the parametric condition effect (referred to as the ‘linear effect’): the linear increase across Categorize, Select Relevant, and Select Both trials. Next, we examined the difference between trials requiring WM and all other trials within the WM blocks (referred to as the ‘WM effect’): all Select Both trials (faces and scenes) + relevant Select Relevant trials (faces in Select Faces blocks and scenes in Select Scenes blocks) – irrelevant Select Relevant trials (scenes in Select Faces blocks and faces in Select Scenes blocks).

A two-step registration process was applied using FSL 4.1′s FLIRT module for linear registration. EPI functional images were first registered to the high-resolution structural image (7 degrees of freedom), then the structural image was registered to standard MNI152 space (12 degrees of freedom). These transformation matrices were combined to provide the transform from EPI to MNI space, which was applied to the results from the above analyses.

Data were combined across runs for each participant using a fixed-effects model, and then modeled using mixed effects at the group level with FEAT's FLAME model (Stage 1 only). Outlier de-weighting was performed using a mixture modeling approach [24]. Results were thresholded at a whole-brain level using cluster-based Gaussian random field theory, with a cluster-forming threshold of z > 2.3 and a whole-brain corrected cluster significance level of p <.05.

### Selection of Regions of Interest

**Figure 2:**
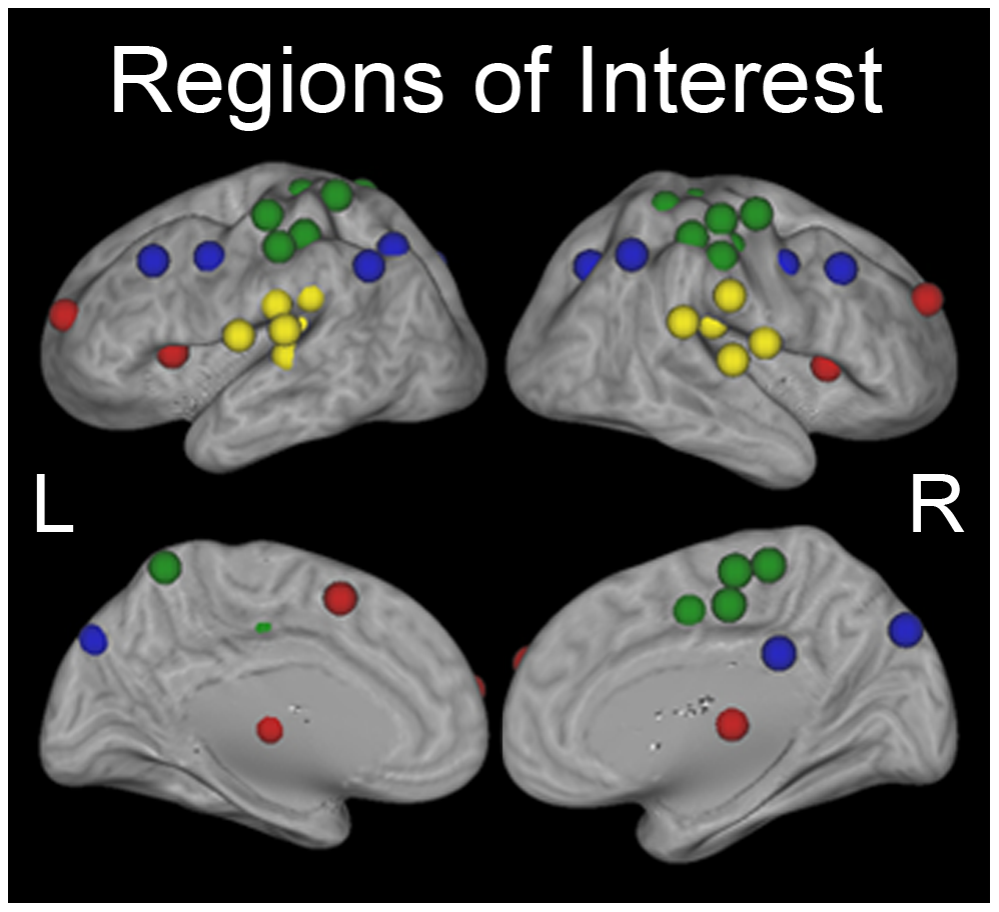
The 49 intrinsic regions of interest (ROIs) utilized in this study. ROIs were defined from two cognitive control networks [7] and two non-cognitive control networks [25]. Red spheres (7) are regions of the cingulo-opercular (CO) network, blue spheres (11) are regions of the fronto-parietal (FP) network, green spheres (18) are regions of the hand somatosensory-motor (SM) network, and yellow spheres (13) are regions of the auditory (AU) network.

Intrinsic network regions of interest (ROIs) were taken from four separate networks identified during rest utilizing graph theoretical techniques and as reported by Dosenbach and colleagues [7] and Power and colleagues [25]. Our analyses focused on the ROIs of the CO and FP cognitive control networks [7]. To test whether results were specific to the cognitive control networks, we additionally included ROIs from two non-cognitive control networks [25]: a task-related network (the hand somatosensory-motor network [SM]) and a non-task-related network (the auditory network [AU]) (Figure 2; Table 1). The original CO and FP ROI coordinates were reported in Talairach space; thus they were transformed to MNI space using the Matlab function tal2 mni.m for the current study (the other network ROI coordinates were originally reported in MNI space). Intrinsic ROIs were created by defining 6 mm radius spheres around the center MNI coordinates of each of the ROIs. The CO network consisted of seven ROIs distributed throughout the anterior prefrontal cortex, anterior cingulate cortex, anterior insula/frontal operculum, and thalamus. The FP network consisted of eleven ROIs distributed throughout dorsal prefrontal cortex, midcingulate cortex, intraparietal regions, and precuneus. The originally-reported SM network consisted of 30 ROIs; due to a slightly limited field of view in the data analyzed here, 12 ROIs that were not within the functional data for all participants were not included, leaving 18 ROIs distributed throughout supplementary motor cortex, precentral gyrus, postcentral gyrus, and superior parietal cortex. The AU network consisted of 13 ROIs distributed throughout temporal cortex, ventral parietal cortex, and parietal and occipital operculum.

**Table 1:** Anatomical region (left) and MNI coordinates in mm (right) of the center of each of the intrinsic network ROIs.

Cingulo-opercular		Fronto-parietal		Hand Somato-sensory-motor		Auditory	
L aPFC	−28, 52, 19	L dlPFC	−43, 21, 38	L PCun	−7, −52, 61	R pI	32, −26, 13
R aPFC	27, 50, 28	R dlPFC	43, 21, 38	L PrCG	−14, −18, 40	R pSTG	65, −33, 20
dACC	−1, 8, 51	L dFC	−41, 1, 39	PCC/midCing	0, −15, 47	R vPlTemp	58, −16, 7
L aI/fO	−35, 14, 6	R dFC	41, 1, 39	R SMA	10, −2, 45	L dPlTemp	−38, −33, 17
R aI/fO	36, 16, 5	midCing	0, −31, 31	L PoCG	−54, −23, 42	L dPlTemp	−60, −25, 14
L aThal	−12, −16, 67	L IPL	−52, −54, 36	L PrCG	−40, −19, 54	L vPlTemp	−49, −26, 5
R aThal	10, −16, 8	R IPL	52, −51, 43	R PoCG	29, −39, 59	R pO	43, −23, 20
		L IPS	−31, −63, 42	R PoCG	50, −20, 42	L pO	−50, −34, 26
		R IPS	30, −65, 39	R PrCG	20, −29, 60	L cO	−53, −22, 23
		L PCun	−9, −76, 36	R PrCG	44, −8, 57	L cO	−55, −9, 12
		R PCun	10, −73, 39	L SPL	−29, −43, 61	R cO	56, −5, 13
				L PoCG	−45, −32, 47	R SG	59, −17, 29
				L PoCG	−21, −31, 61	L pI	−30, −27, 12
				R PrCG/PoCG	42, −20, 55		
				mPrCG	2, −28, 60		
				mPrCG	3, −17, 58		
				R PrCG	38, −17, 45		
				R PoCG	47, −30, 49		

Each ROI was a 6 mm sphere surrounding the center coordinate as reported by Dosenbach and colleagues [7] and Power and colleagues [25]. L  =  left, R  =  right, aPFC  =  anterior prefrontal cortex, dACC  =  dorsal anterior cingulate cortex, aI/fO  =  anterior insula/frontal operculum, aThal  =  anterior thalamus, dlPFC  =  dorsolateral prefrontal cortex, dFC  =  dorsal frontal cortex, midCing  =  midcingulate cortex, IPL  =  intraparietal lobule, IPS  =  intraparietal sulcus, PCun  =  precuneus, PrCG  =  precentral gyrus, PCC  =  posterior cingulate cortex, SMA  =  supplementary motor area, PoCG  =  postcentral gyrus, SPL  =  superior parietal lobule, mPrCG  =  medial precentral gyrus, pI  =  posterior insula, pSTG  =  posterior superior temporal gyrus, vPlTemp  =  ventral planum temporale, dPlTemp  =  dorsal planum temporale, pO  =  parietal operculum, cO  =  central operculum, SG  =  supramarginal gyrus.

### Multivariate Functional Connectivity Analysis

To calculate the task-related functional connectivity specifically for each condition of interest, we implemented a beta-series correlational analysis [26] using least squares estimation (as described by Mumford and colleagues [27]). Briefly, we modeled each event of interest with a separate GLM with two regressors: 1) the event and 2) all other trials and all nuisance regressors. This resulted in a parameter estimate (beta value) for each trial that was robust to collinearity caused by trials in close proximity to each other. An average beta value across all voxels within each intrinsic ROI was calculated, and all average beta values were temporally sorted across all events for a given condition (i.e., correct Select Both trials). A correlation between the sorted beta values (a beta-series) for each pair of ROIs was calculated, resulting in a 49×49 connectivity matrix for each condition of interest. The correlation coefficients were standardized into z-scores in order to allow for statistical conclusions to be made from the magnitudes of the correlations.

To determine average network connectivity, all correlations within each network (referred to as within_CO_, within_FP_, within_SM_, and within_AU_ functional connectivity) and involving ROI pairs that spanned two networks (referred to, for example, as between_COFP_ or between_FPSM_ functional connectivity) were averaged. For between-network connectivity, all possible pairs of ROIs that included regions from two different networks were averaged. So as to not artificially inflate average connectivity due to proximity, the correlation between any pair of regions within 20 mm from each other was not included in any of the averages [25].

Because the CO and FP networks in which we were interested are hypothesized to be cognitive control networks, we focused our analyses on trials with the highest cognitive control demands (Select Both face/scene trials). We also probed the trials with minimal cognitive control demands (Categorize face/scene trials) as a non-cognitive control comparison.

## Results

### Overlap between Group Activation Maps and Intrinsic Networks

**Figure 3:**
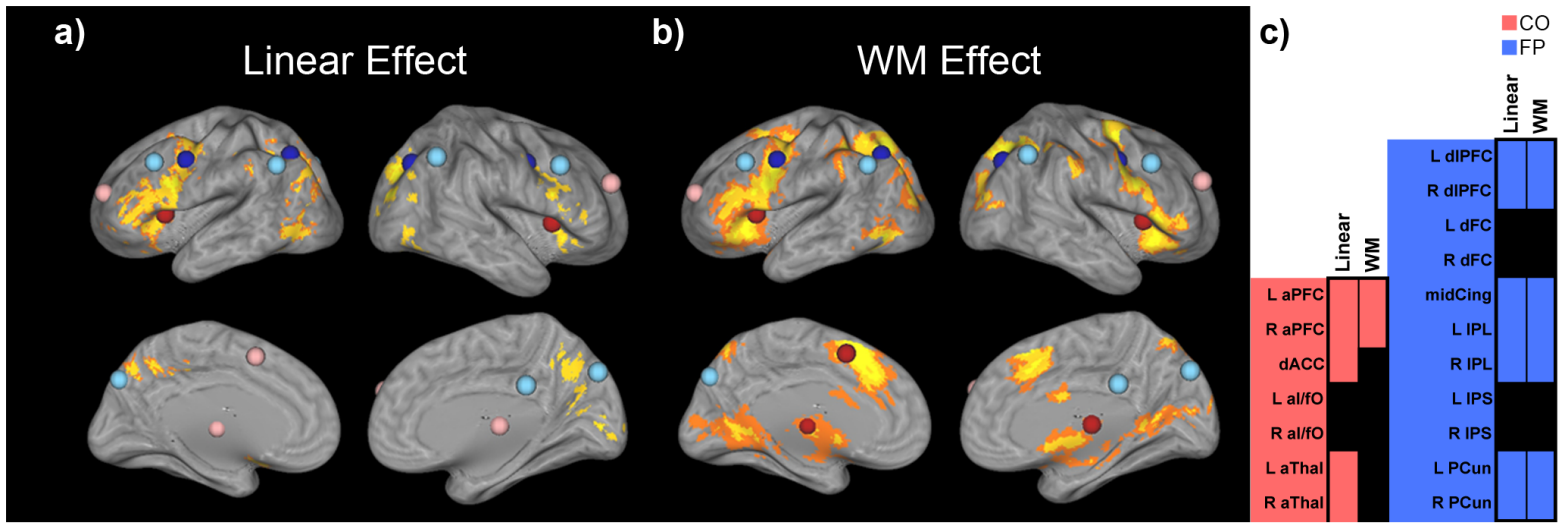
Univariate group maps of cognitive control-related contrasts during the Selective WM task. a) Linear effect (Categorize trials < Select Relevant trials < Select Both trials); b) WM effect (all relevant trials – all irrelevant trials). Overlaid on the group maps are the intrinsic CO (in red/pink) and the intrinsic FP (in blue/light blue) ROIs. Red and blue ROIs depict intrinsic ROIs that overlapped with significant activity related to the contrasts. Pink and light blue ROIs depict intrinsic ROIs that did not overlap with significant activity related to the contrasts. c) Overlap between univariate group maps and intrinsic ROIs (CO ROIs shaded in red; FP ROIs shaded in blue; black squares indicate overlap).

First, we conducted univariate group analyses to determine whether the regions comprising the CO and FP intrinsic cognitive control networks were involved in a task that engages WM. Since our aim was to determine the role of these networks in cognitive control, we conducted two group analyses: the first focused on regions whose activity increased linearly with increased cognitive control demands (linear effect; Categorize trials < Select Relevant trials < Select Both trials) and the second contrasted all trials requiring WM with all other trials (WM effect; all relevant trials [in Select Both and Select Relevant blocks] – all irrelevant trials [in Select Relevant blocks]) (Figure 3a,b). Both contrasts showed similar significant group maps, although, as expected, there were some contrast-specific differences, including differences in extent, as well. This indicates that similar regions are involved in different aspects of cognitive control during the Selective WM task. To examine how each group map related to the intrinsic CO and FP networks, we focused our analyses on the conjunction between the two. The linear effect involved 2 CO ROIs (in the anterior insula/frontal operculum) and 4 FP ROIs (in the dorsal frontal cortex and intraparietal sulcus). When assessing the WM effect, the same 2 CO ROIs, as well as 3 others (in the anterior cingulate cortex and thalamus), and the same 4 FP ROIs were engaged (Figure 3c). Therefore, despite evidence that the CO and FP cognitive control networks are dissociable at rest [7], [28], there was engagement of regions from both intrinsic networks during both cognitive control contrasts. This result is in line with previous research demonstrating the co-activation of regions in the CO and FP networks during a range of cognitive tasks [29]–[31].

### Task-Related Connectivity of Intrinsic Networks

**Figure 4:**
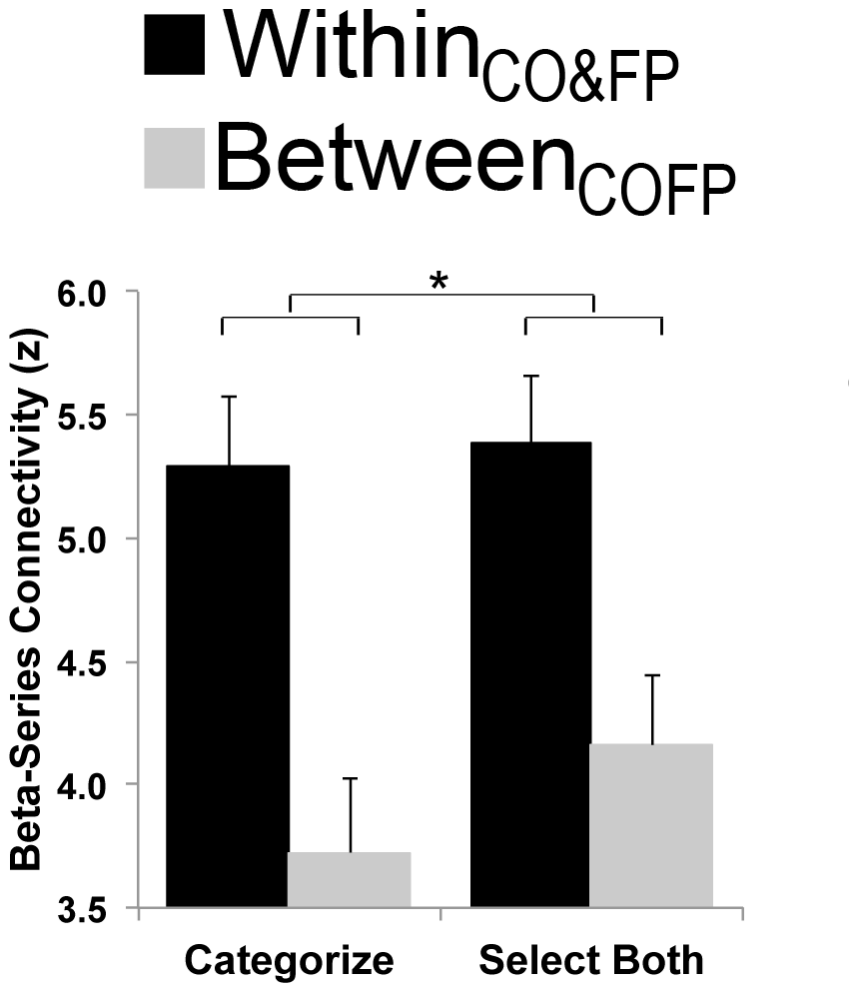
Average within_CO&FP_ and between_COFP_ network connectivity during the Selective WM task. A comparison of network connectivity during Categorize (minimal cognitive control) and Select Both (high cognitive control) trials revealed that while average within_CO&FP_ connectivity did not change, the relative contribution of average between_COFP_ connectivity increased with increased cognitive control demands.

To examine network reconfiguration during cognitive control, we took advantage of the beta-series correlation method [26], [27]. This allowed us to extract connectivity information specifically from individual trials with the highest and the lowest cognitive control demands. We calculated average connectivity within and between the intrinsic CO and FP networks during task performance. Since our aim was to investigate the relationship between within-network and between-network connectivity, we compared total within_CO&FP_ connectivity (the average of within_CO_ connectivity and within_FP_ connectivity) and between_COFP_ connectivity. This allowed us to determine whether there was a difference in overall within- versus between-network connectivity during trials with high cognitive control demands (Select Both trials) as compared to trials with minimal cognitive control demands (Categorize trials). We found greater within- as compared to between-network connectivity for Select Both (t(38) = 2.15, corrected p <.038) and for Categorize (t(38) = 5.41, corrected p <.0001) trials, FDR-corrected for 2 comparisons. Critically, when directly comparing within_CO&FP_ – between_COFP_ for Select Both as compared to Categorize trials, we found that the difference significantly decreased during Select Both trials (paired t(76) = 2.32, p  = .02; Figure 4). In other words, during conditions with high cognitive control demands, between-network connectivity significantly increased relative to within-network connectivity. This pattern of results was specific to the cognitive-control networks: there was no increase of between- relative to within-network connectivity for any other pair of networks during cognitive control (COSM, COAU, FPSM, FPAU, or SMAU; all ps >.20).

### Relationships between Task-Related Connectivity and Performance

**Figure 5:**
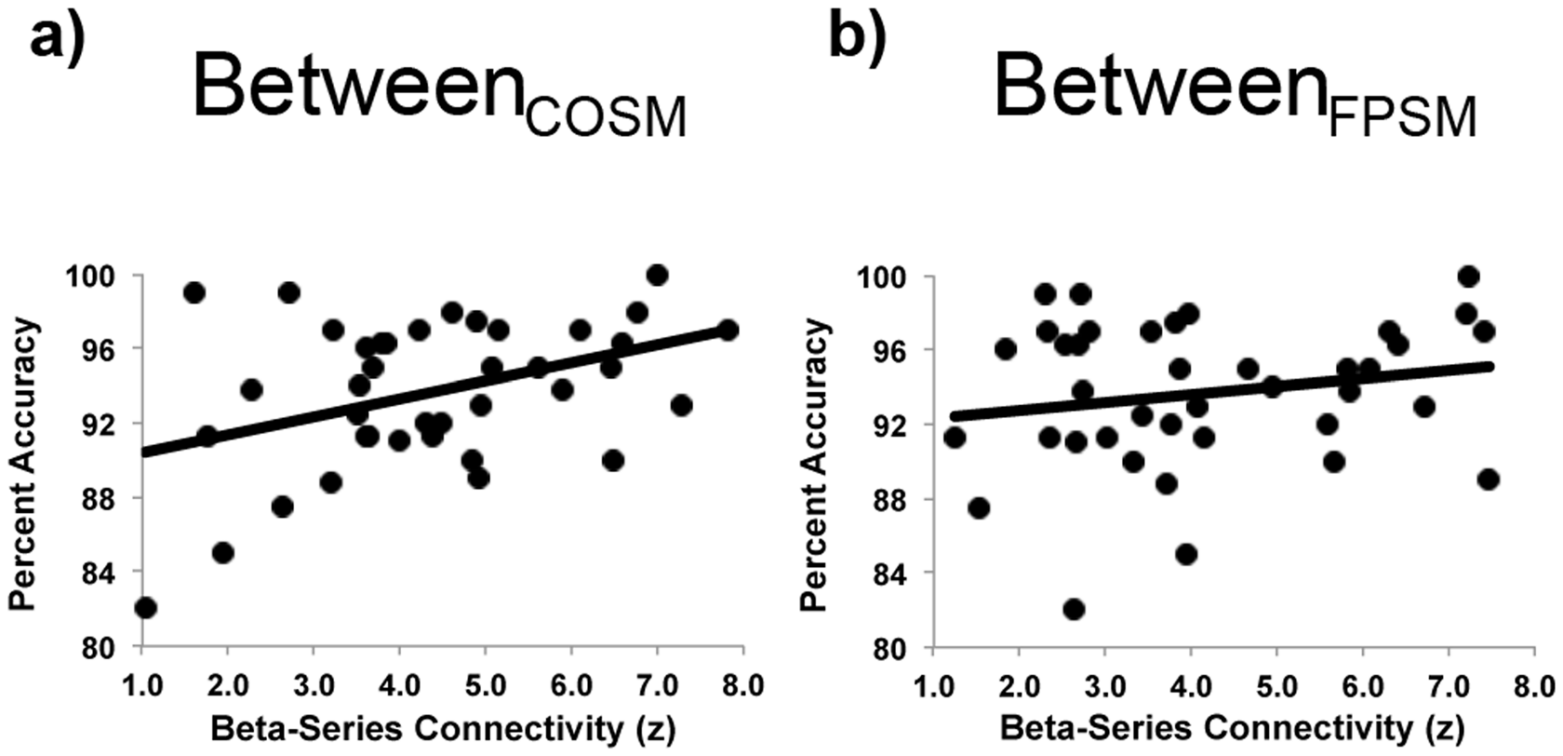
Correlations between accuracy and network integration during Select Both trials. a) There was a significant positive relationship between Select Both accuracy and between_COSM_ connectivity (r = 0.40, corrected p  = .03). b) There was a strong trend toward a similar positive relationship between Select Both accuracy and between_FPSM_ connectivity (r = 0.32, corrected p  = .07).

Last, we examined the relationship between task-related functional connectivity of the intrinsic networks and task accuracy. Once again, we focused on the task conditions with the highest cognitive control demands (Select Both trials). Because we found that between-cognitive control network connectivity increased during Select Both trials, we limited our analyses to between-network connectivity of task-relevant networks (FDR-corrected for three multiple comparisons). While between_COFP_ network connectivity and accuracy were not correlated (corrected p  = .29), participants who were more accurate had greater connectivity between each cognitive control network and the non-cognitive control, task-related network (significant between_COSM_ connectivity and accuracy correlation: r = 0.40, corrected p  = .03; strong trend between_FPSM_ connectivity and accuracy correlation: r = 0.32, corrected p  = .07; Figure 5a,b). This relationship was specific to connectivity with the hand somatosensory-motor task-related network. Correlations relating accuracy to connectivity with the non-task-related auditory network (between_COAU_ and between_FPAU_) were non-significant (both ps corrected for two post-hoc comparisons >.12).

## Discussion

The goal of this study was to examine how the intrinsic CO and FP networks reconfigured during cognitive control and how changes in network organization were beneficial to performance. We found that the intrinsic cognitive control networks became more integrated with each other during conditions of increased cognitive control demands (i.e., functional connectivity between regions spanning the two networks increased) and, crucially, that increased integration between the cognitive control networks and other functional networks involved during cognitive control (i.e., functional connectivity between regions spanning each of the cognitive control networks and the non-cognitive control, task-related SM network) was related to successful cognitive control.

First, consistent with previous results from fMRI studies of resting state data [7], [15], [28], we observed that the intrinsic CO and FP networks were functionally connected in our participants, and that within-network connectivity was significantly higher than between-network connectivity, validating the existence of these networks in our participants.

We also observed that the Selective WM task engaged regions in both the intrinsic CO and FP networks, a result consistent with the literature concluding that these networks are involved in cognitive control [7], [29], [32] and are typically co-activated during cognitive control tasks [30], [31]. Critically, while two different cognitive control contrasts engaged a different subset of CO and FP ROIs, there was a high degree of overlap and both contrasts engaged regions that spanned both intrinsic networks. There is evidence that these are distinct networks at rest [7], and lesion work suggests that they may even operate independently [28]. However, during performance of our WM task when a high level of cognitive control was required, both networks were not only involved [30], but interacted with each other more so than during a condition with minimal cognitive control demands [15].

While within_CO&FP_ connectivity was consistently higher than between_COFP_ connectivity during all task blocks, the difference was smaller during trials requiring the greatest cognitive control. This finding indicates that greater integration between distinct cognitive control networks occurred during increased cognitive control demands. Further, relating connectivity strength to behavior revealed that the magnitude of integration between each cognitive control network and the non-cognitive control, task-related network (SM) was critical for successful task performance. Participants who were more accurate on trials with high cognitive control demands (Select Both trials) displayed greater integration between the CO and SM networks, and between the FP and SM networks. This was despite the fact that between_COSM_ and between_FPSM_ connectivity did not increase during Select Both trials relative to Categorize trials. This may be because while increased CO-FP integration is a universal component of cognitive control (i.e., it increases in all participants), increased integration between cognitive control networks (CO and FP) and non-cognitive control, task-related networks (i.e., SM) is a key mechanism underlying individual differences in cognitive control ability. It should be noted that while the magnitude of the between_COSM_ connectivity correlation with accuracy was numerically larger than the magnitude of the between_FPSM_ connectivity correlation with accuracy, the correlations were not significantly different from each other (z = 0.92, p  = .36). These results imply that not only is communication among cognitive control networks important for WM, but communication between cognitive control networks and other task-related networks, such as the SM network in the Selective WM task, is critical as well.

This increased integration underlying increased performance has been previously observed, with greater integration across the entire brain underlying increased IQ across individuals [9], increased behavioral performance on a continuous performance task [33], and increased speed on an N-back WM task [20]. This study complements and extends those findings in two ways. First, it demonstrates that this observed increase in integration may be localized to task-relevant networks, given that integration with a non-task-related network (AU) was not increased with increased cognitive control demands, nor was it related to performance. Prior results indicating increased global integration related to better performance may therefore have been driven by changes in integration specific to cognitive control and other task-related networks. Second, it implies that intrinsic cognitive control networks, detected at rest, play a core role in the actual execution of cognitive control, since during cognitive control both a selective increase in integration between the CO and FP cognitive control networks, as well as a positive relationship between CO/FP integration with the non-cognitive control but task-related SM network and performance, was observed. While the current study cannot speak to the directionality of these results, future research should examine whether the cognitive control networks cause changes in non-cognitive control, task-related networks during cognitive control, and if that causality influences cognitive control performance.

These findings are consistent with “workspace” theories that propose that better performance on cognitively demanding tasks requires the brain to transiently become more globally efficient (more integrated), even though it is more costly metabolically (i.e., it takes more energy to send information across longer connections), as compared to rest or less cognitively-demanding environments [19], [20]. Such rapid alterations in functional connections in response to changes in cognitive demands have been theorized to be a crucial aspect of cognition [1], [2]. We have demonstrated that not only is increased integration, as measured by functional connectivity using fMRI, observed in situations with increased cognitive control demands, but that intrinsic cognitive control networks are a core aspect of this integration.

In conclusion, we have provided evidence that reconfiguration of intrinsic cognitive control networks occurs in an adaptive manner so as to address current cognitive demands, as reflected in relationships with successful performance. Importantly, our results directly support the assumption that both the intrinsic CO and FP networks underlie cognitive control during task performance. Future work exploring the mechanisms that explain how these networks interact during cognitive performance, both with each other and with other intrinsic networks, and whether they cause task-specific changes in other networks, will be useful in gaining a more complete view of how the intrinsic brain reconfigures to adapt to one's current environment.
